# Methotrexate-induced Hypersensitivity Pneumonitis appearing after 30 years of use: a case report

**DOI:** 10.1186/s13256-017-1333-0

**Published:** 2017-06-28

**Authors:** Mashal Salehi, Robertha Miller, Myint Khaing

**Affiliations:** 1 Department of Medicine, NYC Health and Hospitals/Harlem, Columbia University, New York, USA; 2 Department of Internal Medicine, NYC Health and Hospitals/Harlem, Columbia University, New York, USA

**Keywords:** Hypersensitivity pneumonitis, Methotrexate, Rheumatoid arthritis

## Abstract

**Background:**

Methotrexate has been implicated in a variety of lung complications, one of which is hypersensitivity pneumonitis. Hypersensitivity pneumonitis most often occurs within the first year of starting low-dose orally administered methotrexate. We present a case of methotrexate-induced hypersensitivity pneumonitis after 30 years of methotrexate use, which is the first case to be reported so far.

**Case presentation:**

A 77-year-old African American woman with a history of rheumatoid arthritis presented with progressively worsening shortness of breath and nonproductive cough. She was on a daily dose of 2.5 mg of methotrexate that had been orally administered for the last 30 years. A physical examination was significant for fever of 38.2 °C (100.8 °F), tachycardia, bilateral basal crackles, and oxygen saturation of 88% on room air. A laboratory work up was significant for normal white blood cell count, increased eosinophil count of 18.3%, and erythrocyte sedimentation rate of 111 mm/hour. Sputum cultures were negative for any bacterial pathogens including acid-fast bacilli. Influenza and respiratory syncytial viral infection were ruled out. A (1-3)-B-D-glucan assay (Fungitell®) was within normal limits. Pulmonary embolism was ruled out and echocardiography was normal. A chest X-ray showed hazy opacity with prominent reticulation within the upper lung fields bilaterally, right greater than the left with no pleural effusion. Lung computed tomography revealed nonspecific bilateral upper lung opacification. A pulmonary function test was significant for no obstruction, normal maximum voluntary ventilation, and no restriction, with mildly decreased diffusion. Methotrexate was stopped, and our patient was started on prednisone 60 mg orally administered daily with dramatic clinical and radiologic improvement.

**Conclusions:**

Methotrexate-induced hypersensitivity pneumonitis usually occurs in the initial few weeks to months of starting treatment with methotrexate; however, it can occur late during therapy too, and prompt diagnosis is crucial as it is a reversible condition when diagnosed early.

## Background

Methotrexate (MTX) is a widely used drug for many ailments including psoriasis, rheumatoid arthritis, and cancer. Its benefits are many, but in some the risks unfortunately outweigh the benefits. This case demonstrates that these risks persist despite years of apparent tolerance.

## Case presentation

A 77-year-old African American woman with a history of hypertension, rheumatoid arthritis, and hypothyroidism, presented with progressively worsening shortness of breath of over 1 month’s duration and nonproductive cough. At the time of presentation, she had dyspnea with minimal exertion and need in assistance with activities of daily life. She denied any subjective fever, chills, cough, sputum, hemoptysis, chest pain, palpitation, leg swelling, weight loss, or loss of appetite. She had no history of any contacts with sick people. She had no history of any known allergies and no recent exposure to hay, dust, or toxic fumes. She did not have any pets at home. Her rheumatoid arthritis was well controlled on MTX 2.5 mg administered orally which she took daily for over 30 years. Her vital signs at presentation were notable for an initial temperature of 38.2 °C (100.8 °F), blood pressure of 116/57 mmHg, heart rate of 106 beats/minute, respiratory rate of 16 breaths/minute, and oxygen saturation of 88% on room air. A laboratory work up showed a white blood cell (WBC) count of 9400/uL, with neutrophil percentage of 69.8%. Of significance was an elevated eosinophil percentage of 18.3%. Her erythrocyte sedimentation rate (ESR) was 111 mm/hour. Brain natriuretic peptide (BNP) was within normal limits; her serum albumin was 2.9 g/dL and her lactate dehydrogenase (LDH) level was 265 U/L.

**Fig. 1:**
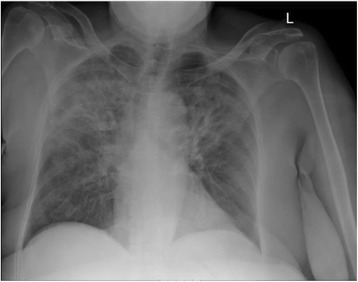
Patient’s chest X-ray on admission

**Fig. 2:**
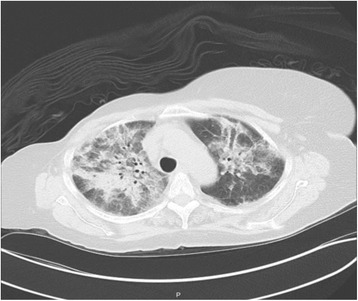
Patient’s chest computed tomography on admission

**Fig. 3:**
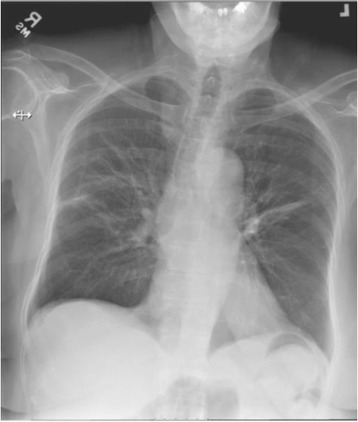
Patient’s chest X-ray after stopping methotrexate and starting steroids

**Fig. 4:**
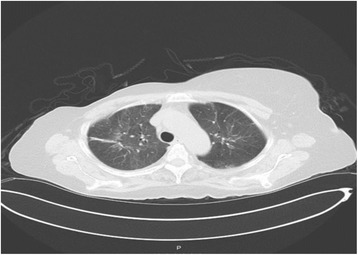
Patient’s chest computed tomography after stopping methotrexate and starting steroids

A chest X-ray (CXR) showed hazy opacity with prominent reticulation within the upper lung fields bilaterally (Fig. 1). Lung computed tomography (CT) revealed nonspecific bilateral upper lung opacification (Fig. 2). Influenza and respiratory syncytial virus (RSV) results were negative. Sputum cultures were negative for any bacterial pathogens including acid-fast bacilli. A urine *Legionella* antigen test was found negative. A (1-3)-B-D-glucan assay (Fungitell®) was within normal limits. Two sets of blood cultures were negative. Echocardiography was normal. A pulmonary function test (PFT) was significant for no obstruction, normal maximum voluntary ventilation and no restriction, with mildly decreased diffusion. She was initially started on empiric antibiotics, MTX was discontinued, and she was started on prednisone 60 mg orally administered daily. One week later, she had significant clinical and radiological improvement with repeat CXR and CT imaging, showing near total resolution of the first noted airspace disease (Figs. 3 and 4). Her eosinophil count went down from 18.3% on admission to 1.1% after treatment with steroids.

## Discussion

MTX pneumonitis is most frequent within the first year of treatment [1, 2] and the reported incidence of this adverse reaction varies from 0.86 to 6.9% [1, 2].

The variable incidence and usual occurrence within a year of starting MTX suggests that pneumonitis is an idiosyncratic immune reaction rather than a dose-related toxic insult to the lung [1, 2]. A review of the literature shows that there are some risk factors that increase the incidence of MTX-induced lung toxicity, including age >60 years, hypoalbuminemia, diabetes, initiation of a second rheumatoid arthritis medication, and daily dosing as opposed to weekly [3]. However, the exact underlying mechanism involved in the disease pathogenesis, and the mechanism by which some of the above factors may increase the lung toxicity risk remains unclear. MTX can compromise the immune response and increase the risk for opportunistic infections due to *Pneumocystis jirovecii*, cytomegalovirus, varicella-zoster virus, *Nocardia*, mycobacteria, or other fungi [4]. There is no diagnostic test that will absolutely confirm the diagnosis of MTX-induced pneumonitis; however, there are some tests that will help in the inclusion or exclusion of other processes. Radiographic evaluation, a trial of drug cessation, bronchoalveolar lavage, and lung biopsy are the primary ways to narrow down the differential diagnosis. However, bronchoscopy and lung biopsy are not necessary in most patients. A beneficial response to MTX withdrawal in the appropriate clinical setting may be sufficient in a patient who is not acutely or severely ill [4]. Up to 50% of patients with subacute onset of MTX lung toxicity demonstrate peripheral eosinophilia, which strongly supports the diagnosis when present [5]. Given the absence of a definitive diagnostic test, a set of diagnostic criteria has been proposed to establish a diagnosis of MTX-induced lung injury [6, 7] which includes: major criteria: (1) hypersensitivity pneumonitis by histopathology without evidence of pathogenic organisms; (2) radiographic evidence of diffuse pulmonary ground glass or consolidative opacities; and (3) blood cultures (if febrile) and initial sputum cultures (if sputum is produced) that are negative for pathogenic organisms and minor criteria: (1) shortness of breath for less than 8 weeks; (2) nonproductive cough; (3) oxygen saturation of less than or equal to 90% on room air at the time of initial evaluation; (4) diffusion capacity of the lung for carbon monoxide (DLCO) less than or equal to 70% of that predicted for age; and (5) leukocyte count of less than or equal to 15,000 cell/mm^3^. MTX pneumonitis is characterized as “definite” if major criteria 1 or 2 and major criterion 3 are present in conjunction with three of the five minor criteria. Probable MTX pneumonitis is present if major criteria 2 and 3 plus two of the five minor criteria are present. However, the clinical utility of the scoring system has not been adequately validated, and it should not be strictly relied upon to establish the diagnosis in each patient [6, 7].

Our patient presented with shortness of breath of less than 8 weeks’ duration associated with a nonproductive cough, an oxygen saturation of less than 90% on room air, with leukocyte count of less than 15,000, a positive radiographic finding, and negative initial blood and sputum cultures.

If one relies on the above diagnostic criteria, then our patient has a “definite’ diagnosis of MTX pneumonitis.

The treatment of MTX-induced lung toxicity involves discontinuation of MTX, empiric antimicrobial therapy at likely pathogens until the definitive procedures and cultures are performed, and high-dose glucocorticoids. However, there has been no optimal therapy established yet and no perspective trial of therapies has been performed [4].

In our patient, as expected the differential diagnosis was significantly broad, including bacterial, viral, and fungal infections and rheumatoid arthritis-related interstitial lung disease, besides MTX-induced lung toxicity and many others,

The combination of dyspnea, a history of MTX intake, upper lobe infiltrates on imaging studies and, most important, eosinophilia led us to consider MTX-induced lung toxicity on the top of our differential diagnosis. The same findings plus the absence of a restrictive pattern on pulmonary function tests makes the possibility of rheumatoid arthritis-related interstitial lung disease less likely.

There was no history of a recent exposure to hay, dust, or toxic fumes that would suggest these as the underlying cause for our patient’s findings.

Among the viral etiologies, influenza and RSV were ruled out. Viral etiologies in immunocompetent patients are usually self-limited ﻿and tend to improve over time. On the other hand, in immunosuppressed patients like ours, they can present as a serious clinical condition and usually need antiviral treatment before any improvement is seen.

Our patient did not have any other signs or symptoms of acute viral infection besides cough and shortness of breath, and there was no symtopatic improvment over time which is typically seen in any infection of viral etiology. This plus her excellent response to steroids instead of any antiviral therapy made viral etiologies less likely. Furthermore, In viral etiologies, the addition of steroids may worsen the patient’s clinical condition by further lowering their immunity which was not the case in our patient; also, eosinophilia is not seen in viral infections. The patient's (1-3)-B-D-glucan assay (Fungitell and LDH results were within normal limits which makes a fungal etiology less likely too. In summary, our patient's prompt clinical and radiologic response to MTX withdrawal and steroids administration with a reversal of eosinophillia, excluded rheumatoid-related interstitial lung disease, viral, bacterial and fungal etiologies.

## Conclusions

Our case report shows that although hypersensitivity pneumonitis has been reported to occur after initial weeks or months of starting the medications, it can occur even after 30 years of use. Clinicians should be aware of this fact and keep hypersensitivity pneumonitis in their differential diagnosis as it is a reversible disease if diagnosed early.

Although there are no definite diagnostic criteria for establishing the diagnosis of MTX-induced lung toxicity, the best approach would be to combine the clinical, laboratory, and radiologic findings together to have an appropriate management plan. This would include ruling out acute infections, a trial of MTX discontinuation, and treatment with high-dose steroids, especially when a patient is not severely sick. The decicison to perform a bronchoscopy or lung biopsy should be made on an individual patient basis.

## Abbreviations

BNP: Brain natriuretic peptide
CT: Computed tomography
CXR: Chest X-ray
DLCO: Diffusion capacity of the lung for carbon monoxide
ESR: Erythrocyte sedimentation rate
LDH: Lactate dehydrogenase
MTX: Methotrexate
PFT: Pulmonary function test
RSV: Respiratory syncytial virus
WBC: White blood cell.
